# Grasping the meaning of perinatal palliative care for the multiprofessional team

**DOI:** 10.1590/1984-0462/2024/42/2023178

**Published:** 2024-05-27

**Authors:** Fernanda Pegoraro de Godoi Melo, Adriana Valongo Zani, Juliane Pagliari Araujo, Adriana Martins Gallo, Marcelle de Oliveira Peripolli, Vanessa Suziane Probst

**Affiliations:** a Universidade Estadual de Londrina, Londrina, PR, Brasil.; b Universidade Estadual de Maringá, Maringá, PR, Brasil.; c Faculdade Pequeno Príncipe, Curitiba, PR, Brasil.

**Keywords:** Palliative care, Patient care team, Neonatology, Perinatal care, Humanization of care, Cuidados paliativos, Equipe de assistência ao paciente, Neonatologia, Assistência perinatal, Humanização da assistência

## Abstract

**Objective:**

To grasp the meaning of perinatal palliative care for the multidisciplinary team.

**Methods:**

This is a qualitative study guided by content analysis. The study included 56 health professionals working in maternal and child units of a public university hospital. A semi-structured interview was conducted, which was recorded and subsequently fully transcribed. The collection took place from June 2018 to May 2019. Data were entered and exported to Atlas ti: The Qualitative Date Analysis & Research Software, version 23.1.1.0.

**Results:**

Four thematic categories emerged from the data analysis: palliative care and eligible public in the view of professionals; communication between family and team in decision-making; assistance in palliative care; humanized care.

**Conclusions:**

The professionals think of palliative care in Perinatology in a similar way and perceive the difficulties of communication with the family and decision-making. They agree that it is necessary to provide greater support to the family, and to provide comfort measures, either for the non-viable fetus or for the baby eligible for palliative care.

## RESUMO

**Objetivo:**

Apreender o significado dos cuidados paliativos perinatais para a equipe multiprofissional.

**Métodos:**

Trata-se de um estudo qualitativo orientado pela análise de conteúdo. Participaram do estudo 56 profissionais de saúde atuantes em unidades materno-infantis de um hospital universitário público. Foi realizada uma entrevista semiestruturada, a qual foi gravada e posteriormente transcrita na íntegra. A coleta ocorreu no período de junho de 2018 a maio de 2019. Os dados foram digitados e exportados para o *software* Atlas ti: *The Qualitative Date Analysis & Research Software*, versão 23.1.1.0.

**Resultados:**

Quatro categorias temáticas emergiram da análise dos dados: cuidados paliativos e público elegível na visão dos profissionais; comunicação entre família e equipe na tomada de decisão; assistência no cuidado paliativo; cuidado humanizado.

**Conclusões:**

Os profissionais significam os cuidados paliativos em perinatologia de modo semelhante e percebem as dificuldades de comunicação com a família e a tomada de decisão. Concordam que é necessário fornecer maior apoio à família e proporcionar medidas de conforto, seja para o feto inviável, seja para o bebê elegível para cuidados paliativos.

## INTRODUCTION

Technological advances and the development of health sciences have allowed an increase in survival rates of extreme preterm newborns (NB) and/or with severe congenital malformations.^1-3^ However, this has led to an increasing number of children with severe sequelae and physical and intellectual disabilities, increasing and prolonging the suffering of these patients, their families and the caregiver team.^2,3^

These NB survive under care in neonatal intensive care units (NICU), are clinically fragile, technologically dependent,^4^ and the increased life span in these cases has not necessarily caused improvement in quality of life. The limitations of life, such as the finitude of the human being, in some cases, are avoided by health professionals through "therapeutic obstinacy", that is, the use of disproportionate measures to avoid death. The prolongation of suffering should not be adopted as a practice to replace palliative care (PC) in eligible patients.^1^

The World Health Organization (WHO) highlights that the PC seeks to promote the quality of life of patients and family members who face life-threatening diseases, through prevention and relief of suffering, focusing on comprehensive care, and includes early identification, evaluation and treatment of pain. It also helps in problems of a physical, psychosocial and spiritual nature.^5^

In the neonatal area, several conditions are eligible for PC, such as progressive and degenerative diseases, irreversible situations such as severe ischemic encephalopathy, chromosomal abnormalities, severe congenital malformations, extreme prematurity and heart disease.^6^ And for the effective development of this mode of care, care plans should be carried out, with the involvement of the family and multidisciplinary team, to ensure that they continue without interruption or bias, always seeking to improve the quality of life of the sick and their families.^1,3,5,7^ Pregnant women with serious diseases and risks, both for them and for the baby, tend to have to make emotionally challenging decisions about a possible termination of pregnancy, and PC aims to support these families.^8^

In the United Kingdom, the approach in the perinatal care of babies and families in situations of diagnosis or recognition of limiting conditions is aimed at strengthening the professional-parents and interprofessional relationship, valuing communication and also highlighting the need for a care plan that encompasses the baby and the family.^9^ A study conducted in Bangladesh^10^ points to the valuable contribution of PC and the need to encourage discussion on the adaptation of professional work plans based on family and parental needs and preferences. Thus, when integrating PC in humanitarian programs, the PC is emphasized as an important reducer of suffering in its main role.

In the last decade, the field of research related to PC in neonatology was on the rise worldwide.^11^ A recent analysis that aimed to quantify admissions eligible for PC among hospitalizations in neonatal units in England and Wales identified that at least 2% of the babies needed PC proven by the categorical criteria of the British Perinatal Medicine Association, and that most of them survived until the moment of hospital discharge.^12^ In Brazil, the centers that have sections that offer PC have grown gradually in recent years,^13^ and, in 2019, had about 190 registered services, an increase of 8% compared to 2017; still insufficient, however, to place Brazil in the group of countries with better level of coverage in PC.^14^ Moreover, there is still a deficiency in the training of human resources due to the small number of institutions that offer specialized courses.^13^ Given this reality and the amount of NB with severe malformations or that do not respond to the instituted therapy or severe pregnant women with life-limiting conditions, it is necessary to implement PC in the scope of assistance and research to this population group.

In the maternal-child area, parents and NB are currently the focus of attention of PC, but it is necessary to advance in supporting the multidisciplinary team that provides assistance to this population group.^11^ This study is justified by the importance of discussing the PC with the care practice of the multidisciplinary team and intends to answer how the multidisciplinary team perceives the care of pregnant women with fetal abnormalities and the neonates of poor prognosis and their family members. The objective of this study was to grasp the meaning of perinatal palliative care for the multiprofessional team.

## METHOD

Qualitative study guided by content analysis according to Bardin.^15^ In order to maintain methodological rigor, it was conducted based on the support tool Consolidated Criteria for Reporting Qualitative Research (COREQ).^16^

The study included 56 health professionals from different professional categories working in the Maternal and Child service (maternity, obstetric emergency service and Neonatal Intensive Care Unit [NICU]) of a public university hospital located in southern Brazil. This hospital is a highly complex and reference service for the northern region of the state of Paraná (Table 1).

**Table 1:** Numerical distribution of the category of health professionals and their locations/areas of activity.

Professionals		Participants (n=56)
Technical Level		
	Obstetric Emergency Service Nurse Technician	04
	Neonatal ICU Nurse Technician	14
	Maternity Nurse Technician	01
Residents		
	Medicine – Gynecology and Obstetrics	03
	Medicine – Pediatrics	04
	Medicine – Neonatology	05
	Nursing – Gynecology and Obstetrics	01
	Nursing – Neonatology	06
University level		
	Physician – Neonatal ICU	05
	Physician – Obstetrics	02
	Psychologist – Neonatal ICU	01
	Physiotherapist – Neonatal ICU	03
	Nurse – Obstetrics	03
	Nurse – Neonatal ICU	04

ICU: intensive care unit.

Inclusion criteria were professionals with more than six months of experience in the institution and who had experienced the care of pregnant women with fetal abnormalities and neonates with poor prognosis. Employees on vacation, medical leave or maternity leave were not included in the period established for data collection.

Data collection took place from June 2018 to May 2019, through the technique of individual interviews and a semi-structured interview script, containing two parts: the first referring to sociodemographic data and the second referring to the theme. The guiding questions were: What do you consider to be pregnant woman with severe fetal abnormalities and/or neonates with poor prognosis (pathologies, clinical situations)? If there was a situation of a pregnant woman with severe fetal abnormalities and/or a neonate with a poor prognosis in your family, how would you like the treatment and care to be performed?

Initial contact with the participants occurred from the insertion of the main researcher in a WhatsApp group, aimed at employees of the institution who were professionals in the area of maternal and childcare. In this virtual environment, an invitation was made by text message with an acceptance link. Once integrated into the research, the participant was invited to name more people who could contribute, leading to recruitment of study participants through the "snowball" method. When the indicated participant met the inclusion criteria, a meeting was planned with him/her personally or by telephone contact, WhatsApp or email (according to what was available).

The interviews were recorded by digital recorder, and they occurred in the work environment itself, in reserved places chosen by the interviewees, usually in a rest room or pantry attached to the work sector, respecting their availability, privacy, anonymity and interest in participating. Data collection occurred earlier during the working hours of the professionals.

The interviews lasted approximately 30 minutes considering the interaction of the interviewer and the professional and the interview itself. At the end of the interview, participants were able to listen to the recording and confirm and/or change the lines.

The data collection was carried out by six female students of the nursing graduate course, all trained for the collection of qualitative data through simulations of interviews that were conducted by the main researcher and the co-supervisor, a health professional with experience in qualitative research, with a training duration of 24 hours. Such care was taken because the main researcher of the study was part of the team of the maternal and child unit working as a doctor, which could generate bias in the study if she conducted the interviews. The recorded data were transcribed in full for further analysis and archived as provided in the ethical-legal determinations.

The number of participants was not determined at the beginning of the study, since the researchers were careful to include professionals of different functions in the Maternal and Child Unit and who are necessary to compose a team of perinatal and neonatal palliative care to obtain a more comprehensive perception of the multidisciplinary team. The researchers took care to interview professionals from all shifts to enable an expanded view of the perception of these professionals. Participants were guided about the objectives of the research, informed that they could give up at any time, that they would not receive any monetary value to participate and that, in case of doubts, they could contact the lead researcher using the contact information provided.

Interruption of collection occurred after the study had encompassed the representation of the professional categories that composed the multidisciplinary team working in the area and that experienced the care of pregnant women with fetal abnormalities and neonates with poor prognosis, which, in qualitative research, is fundamental to determine the interruption of data collection and definition of sample size.^17^

Data were entered and exported to the software Atlas ti: The Qualitative Data Analysis & Research Software, version 23.1.1.0, license L-C6B-06F, for the codification of discourses and subsequent grouping of themes with the participants’ reports on PC in the maternal-child area.

**Figure 1:**
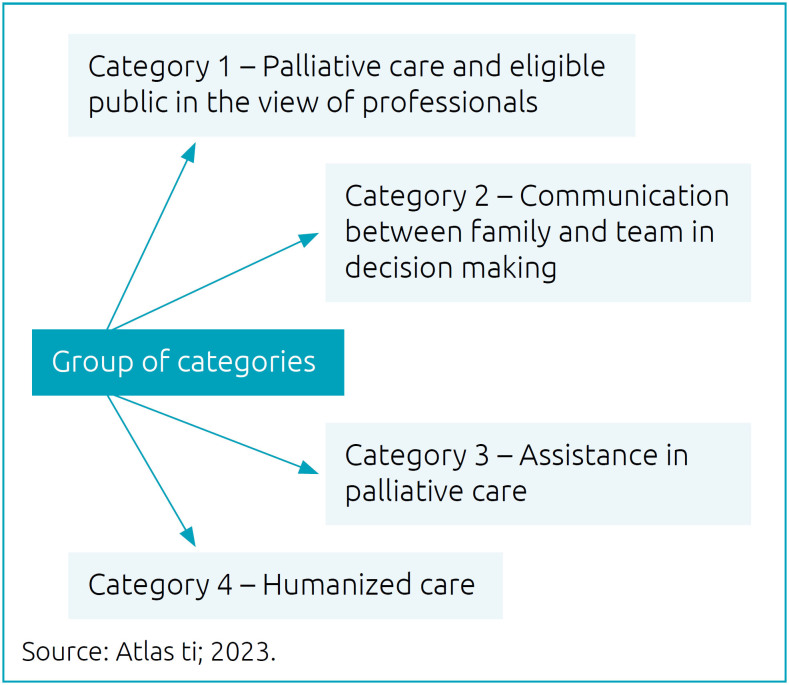
Category coding tree.

Source: Atlas ti; 2023.

Content analysis was used in the thematic modality,^15^ which allows highlighting the core meanings of communication, according to the methodological procedures: data organization, categorization and coding, inference and interpretation of the results. After this analysis, with the help of the Atlas ti software, 197 insertions emerged; 41 encodings and four categories (Figure 1).

The rigor of the research was confirmed through the credibility and thorough care that the researchers took in approaching the data from collection to analysis. This involved transcription of the interviews, identification of the sentences, coding and grouping of codes, with constant comparison of data and triangulation by means of comparison and critical discussion in the research group, among the researchers involved.^18^ This process took place in order to validate the categories that emerged with the study findings.

The project was approved by the Research Ethics Committee under n. 2,568,478 and carried out with an authorization signed by the participants before the beginning of the interviews — the Informed Consent Form (ICF), in two copies, one of which remained with the participant and the other with the interviewer. In order to maintain the anonymity of the participants, their speeches were identified by acronyms, as follows: Obstetric Emergency Service Nursing Technician (TEPSO); Neonatal ICU Nursing Technician (TE); Maternity Nursing Technician (TEMAT); Gynecology and Obstetrics Resident Doctor (MRGO); Pediatrics Resident Doctor (MRPED); Neonatology Resident Doctor (MRNEO); Gynecology and Obstetrics Nursing Resident (REGO); Neonatology Nursing Resident (RENEO); Neonatal ICU Doctor (MNEO); Gynecology and Obstetrics Doctor (MGO); Psychologist (ICU); Neonatal ICU Physiotherapist (FISIO); Obstetric Nurse (ENFOBST); Neonatal ICU Nurse (ENEO). The acronyms were followed by numbers, according to the order of data collection.

## RESULTS

Regarding the characterization of professionals, most were female (91%), the median age was 32.5 [27.3 to 32.5] years, with the highest number of people in the age group between 24 and 59 years (87.5%). Regarding marital status, 46.4% were single. Most professionals have a weekly workload of 40 hours (53.6%). Regarding professional category, most of the interviewees were doctors and nursing technicians (33.9%), followed by nurses (25%). Regarding experience in the field, 60.7% of the participants had less than ten years of experience in the area and 58.9% had a residency as complementary training.

From the data analysis, four thematic categories emerged: palliative care and eligible public in the view of professionals; communication between family and team in decision-making; assistance in palliative care; humanized care.

### Palliative care and eligible public in the view of professionals

The interviews showed that health professionals recognize various syndromes and malformations as eligible for PC in maternal and childcare, highlighting life-threatening syndromes and malformations.

### Communication between family and team in decision-making

*Significant anomalies that end up compromising the proper functioning of their organs (MRPED 4).*

*Malformations incompatible with life or requiring numerous interventions and procedures that do not bring certainty of improvement or cure (RENEO 5).*

*Severe malformations of the Central Nervous System (CNS); heart malformations with poor prognosis; extreme premature neonates < 23 weeks; genetic diseases associated with severe malformations; severe perinatal asphyxia with major CNS injury (MNEO 1).*

The category shows that communication between family and professionals is essential for the care plan to be organized and carried out effectively so that professionals feel safe in exercising their role properly without overloading family members in decision-making. However, sometimes this premise is not considered, hindering inclusion of the family and injuring PC precepts.

*It is important to include the family in the decision making, they have the right to give an opinion on the desired treatment for the child* (TE 1).

*The child is the parents’ most precious thing and they should be aware of and participate in all stages. A reserved environment to talk to parents. The most important thing is that parents feel welcomed and respected by the team. To respect the parents’ moment, the most important thing is a lot of conversation and to let the parents go through all stages: suffering, pain, mourning, denial, acceptance. I think it is important to offer treatment in another service, if applicable* (MNEO 5).

*That the team respected our decisions, clarified doubts, and that everyone spoke the same language* (MRNEO 1).

*It is appropriate for the family to choose which conditions should be taken from the diagnosis. Not to give the family the option "to invest or not to invest" because this should not be a burden that the family will carry if something is not according to plan* (RENEO 4).

*Most of the time we do not ask the family if they want to invest or not, revive or not, our tendency is to do everything there, to do all we can to bring the child back* (ENEO 3).

### Assistance in palliative care

Regarding how some professionals believe that assistance to patients eligible for PC should be conducted, it was evidenced that the health professionals consider that the care plan to be performed during the PC should include the presence of the family, measures of comfort, management of symptoms, as well as more proactive approaches focusing on the relief of suffering and providing impeccable treatment of pain and other physical, psychological and psychosocial problems.

*It would be in line with the absence of extreme measures, resuscitation or invasive procedures. I would just like general care, hygiene and pain relief* (MRNEO 3).

*[…] comfort measures for the patient in order to avoid greater suffering to the patient and family members* (MRNEO 05).

*To offer comfort to him or her while alive rather than insisting on treatments that will only prolong suffering* (RENEO 03).

*If it is palliative, there will be minimal procedures, we will not reanimate or anything, we will only give comfort, we will warm up, we will expect it to evolve to what is inevitable* (MRPED 02).

*It is important to include the family in decision making, they have the right to give an opinion on what treatment they want for the child* (TE 1).

### Humanized care

This category allowed us to infer that the professionals all agree on what procedures would provide humanized PC. According to the interviewees’ speeches, these are aimed at well-being, such as to reduce pain and provide comfort, they are paramount, and attention to the family, respect and dignity are essential to humanize the entire process as appropriately as possible. By seeking comfort and quality of life through symptoms control, more days and quality of life will also be possible.

The humanized approach focuses on the comprehensiveness of the human being. Intervention in symptoms of a physical, social, emotional and spiritual nature transform the practice of PC into a necessary, interprofessional teamwork that seeks to meet the patient and their family.

*[…] to provide comfort, reduce pain, care for her with dignity, with respect, and for the baby too, to continue offering the diet, to do the basics with a humanized look, looking at the baby or the mother with a humanized look, because they are human beings and it is not because they are under palliative care that we have to act differently. We have to keep taking care of them as far as they can go* (TE 6).

*In the most humanized way, which did not exclude the family from the place […]* (ENEO 4).

*[…] it is giving attention to family, if, for example, there is nothing to do indeed, if the child is inviable, I think it is giving psychological support, having a suitable place for the mother suddenly to give birth or abort, with the presence of the family, something related to support of an emotional kind, you know, for her not to feel alone* (TE 10).

Given the categories that emerged in the analysis of the data on "communication between family and team in decision making" and "assistance in palliative care", which point to the interactions, and the category of "humanized care", which points to the consequences of the actions, using the Atlas ti software, it was possible to construct the Sankey Diagram to help visualize these categories and their co-occurrences.

In the diagram, the width of the bands between the subcategories is proportional to the integration and flow of relations between the cross-subcategories, allowing us to infer, based on the respondents’ speech, that there is a relationship between the events experienced/reported and the offer of PC to a greater or lesser degree. The subcategories were highlighted in color, according to the verb used in data analysis. They were grouped on the left side as: green=desire, red=identify; and on the right side as: blue=need and yellow=recognize. The interactions between these subcategories appear in the Sankey diagram cross-referenced, generating, in part of the bands, secondary colors according to the intensity of the correlation.

**Figure 2:**
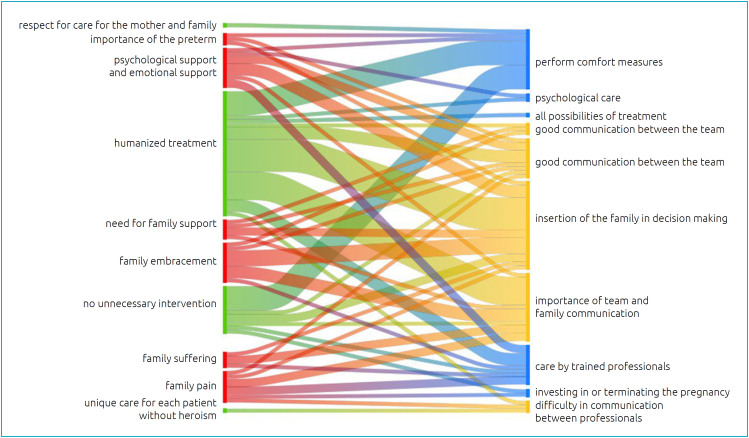
Sankey diagram: intensity of relationships regarding palliative care in the maternal and child area experienced by health professionals.

The subcategories highlight the strong flow of the relationship between "desiring humanized treatment" and the need to "carry out comfort measures", "recognize the importance of family inclusion in decision making" and "communication between team and family". The co-occurrence of these four subcategories and also the interaction between "desiring no unnecessary intervention" and the identification of "performing comfort measures" supports the humanized care category (Figure 2).

## DISCUSSION

In Brazil, the health system still presents high neonatal mortality due to avoidable as well as inevitable situations. In this context, the need for perinatal PC is evident as a means of support in such situations.^13^ For many years, an increasing technicism in health care has favored the view that intensive care therapeutic practices should focus on increasing short-term survival.^1,19^ Some factors contribute to the non-inclusion of PC in the care of the maternal-child population, such as the difficulty of health professionals in understanding the terminality of human life and feelings such as frustration, failure and impotence.^1^

In addition, this contribution highlights the lack of specialized training in PC in Brazil.^17^ Thus, there is a need to deepen and explore formalized programs and health education on PC, in order to provide high-quality assistance to eligible NB and their families.^20^

The consolidation of a multiprofessional PC team is fundamental to achieve the goal of providing comprehensive and quality care, supported by fundamental and necessary pillars for this type of care.^3^ A scenario in which there is little life expectancy for a baby or a young mother^17^ is common for PC professionals in neonatology, and the team's multiple looks aim to meet the patient and their family in all dimensions. In addition to the treatment and necessary interventions, they can ensure well-being, support the mourning of parents, giving psychological, social and spiritual support to the family.^21^ The integrality of this team is fundamental for the success of actions and decision making throughout the care process.^22^

Dialogue is a tool that needs to be present throughout the care process, especially for populations that require specialized care, as it enables the identification of the needs of patients and their families,^23-25^ as well as helping to prepare the family for the process of loss and mourning, reinforcing the human character of caring in particularly difficult situations.^25,26^

Communication of the care performed in palliative patients often enables greater agreement regarding the care plan, when patient and family participation results in joint decision making on the interventions to be performed.^25^

It was identified that the participants of this study present convergent difficulties concerning the communication of common themes in PC, especially in decision making in end-of-life situations and decisions shared with the family. A Swiss study on uncertainty and improbability in neonatal decision making at the end of life showed that the uncertainty that is inherent in the complex process experienced before the problem makes the situation complex. This fact makes it difficult and can even prevent the opportunity to discuss parental values and preferences in the shared decision-making process between team and family.^27^

Thus, PC is challenging from an emotional and professional point of view and can somehow lead to divergent opinions among co-workers, and a Kwait^20^ study showed that these divergences do not affect confidence when performing PC skills. It is important to emphasize that the appropriate interprofessional relationship and the establishment of a dialogue can benefit communication with NB parents in palliative care. This allows the sharing of information about environment, routines, tools and procedures, as well as the appreciation of joint decisions.^3,25,28,29^

Regarding comfort care for neonates, it should be integrated for babies diagnosed in prenatal or postnatal periods with life-limiting conditions.^30^ It is important to control pain and relieve symptoms, providing decreased concern during treatment. One of the principles of this philosophy of care is, in addition to comfort, to provide quality of life and reduce suffering, with a focus on the patient and the family.^30,31^

For effective operationalization of the care plan, the multidisciplinary team needs to be trained to meet the maternal-child population and the family as a whole. This includes minimizing distress, controlling pain, and relieving symptoms, which contributes to a better quality of life regardless of the time remaining.^31^

It is necessary to break the stigma that PC is synonymous with terminal patients. The participants of the research also highlighted the importance of performing humanized care, including the family in it, performing comfort measures and the need for communication between the family and care team, denoting a co-occurrence between these pillars of care. A study conducted in Bangladesh shows that the predominant understanding in relation to pediatric PC was to prioritize comfort for babies and children with short survival expectation; and that the opinions of professionals were influenced by their sense of responsibility to avoid harm, to do the best for patients, and by their religious beliefs in determining the outcome of the child.^10^

Integrating the family in the assistance provided to the pregnant woman and NB in PC should be one of the main focuses, by offering psychosocial and spiritual support and allowing the family bond to be the most frequent component of care. This is because the creation of memories, which is important at times like these, occurs with the constant presence of the family, either in the maternity or in the NICU.^11^

As an implication for the practice, it was possible to understand the perception of the multiprofessional team that acts in maternal and child health on the relevance of PC in this area. This study is unique in that it provides relevant information that will serve as a subsidy for the improvement of PC implementation actions in the maternal and child area, since it includes professionals from various areas of perinatal care. Among the limitations of the study, data were collected in a single health institution and should be expanded to other realities.

The context discussed in this study pointed to a gap which becomes evident in the need for a differentiated and expanded approach in understanding PC. Sometimes, the structuring and planning of care are focused only on the present moment at the hospital, without much discussion of future care, with a view to the vital finitude previously established by deduction and viability. However, as an insight for this study, given the experience of other countries, an approach on future care plans is suggested, regardless of the prognosis, one in which continuity is provided to embrace and care for the patient and their family.

Thus, further research should be developed, deepening this subject of considerable relevance to the area of neonatology, especially from the perspective of pregnant women and/or parents of NB.

We conclude that, regardless of the professional category, palliative care is viewed in perinatology similarly. Professionals recognize the difficulties of communication with the family and in decision making. They agree that greater support to the family is needed, and provide comfort measures either for the unfeasible fetus or for the baby eligible for PC. These results contribute to the planning of interventions that can guarantee perinatal palliative care.

## Data Availability

The database that originated the article is available with the corresponding author.
